# Delivery without Rupture of Hymen—Remarks

**Published:** 1874-11

**Authors:** J. A. Meek

**Affiliations:** Jonesboro, Ark.


### DELIVERY WITHOUT RUPTURE OF HYMEN—REMARKS.

BY J. A. MEEK, M.D., OF JONESBORO, ARK.

*Editor Atlanta Medical and Surgical Journal:*

In looking over the February number of the *Atlanta Medical and Surgical Journal,* on page 650 we find copied from the *Western Lancet* the report of a case of pregnancy and delivery without rupture of the hymen, by R. Beverly Cole, M.D., Professor of Obstetrics and Clinical Diseases of Women, University of California.

I can hardly concede that the Professor's management of this case (although I may not be altogether capable of deciding in such matters) was according to scientific principles.

Professor Cole informs us that he arrived at the house of the patient several hours before delivery. He also states that upon his arrival he instituted an examination, and discovered the hymen in a normal condition—in an unruptured state.

Will the Professor allow us to ask him a question just here? Why did he not at once divide the hymen? He states that, from the first, he was apprehensive of perineal rupture. The rupture was occasioned by the unyielding condition of the hymen. Knowing such results were likely to occur, he certainly must have had good reasons for refusing to divide or rupture the hymen at once. What these reasons were, we are at a loss to determine. It was certainly not from an apprehension of hemorrhage from that source. We are not aware that any hemorrhage is likely to be set up in such an operation. There can be but little difficulty attending the operation, the hymen being so readily reached.

What is still more remarkable in Prof. Cole's management of this case is, that notwithstanding several months had elapsed since the birth of the child, yet no effort, it seems, had been made for the division of the membrane. Prof. Cole informs us that he submitted a proposition to the mother for the closing of the fistulous opening, but does not say one word about dividing the hymen, although, as he states, it still remains intact.

This operation is frequently performed by the merest tyro in medicine in the wilds of Arkansas. I had a student of medicine some two years since who, without hesitation, performed this operation successfully, so far as I know, without the least trouble. Will Prof. Cole submit a supplementary report in this case, giving us his reasons in full for his course in this most remarkable and interesting case?

